# Specific Visualization of Tumor Cells Using Upconversion Nanophosphors

**Published:** 2014

**Authors:** E. A. Grebenik, A. N. Generalova, A. V. Nechaev, E.V. Khaydukov, K. E. Mironova, O. A. Stremovskiy, E.N. Lebedenko, A. V. Zvyagin, S. M. Deyev

**Affiliations:** Shemyakin/Ovchinnikov Institute of Bioorganic Chemistry, Russian Academy of Sciences, Miklukho-Maklaya Str., 16/10, Moscow, 117997, Russia; Lomonosov Moscow State University, GSP-1, Leninskie Gory, Moscow, 119991, Russia; Institute of Laser and Information Technologies, Russian Academy of Sciences, Pionerskaya Str., 2, Troitsk, 142190, Russia; Lobachevsky Nizhniy Novgorod State University, Gagarina Prospekt, 23, Nizhniy Novgorod, 603950, Russia; Department of Physics and Astronomy, Macquarie University, Sydney, NSW 2109, Australia

**Keywords:** upconversion nanophosphors, biomarker imaging, anti-tumor antibodies, self-assembly, HER2

## Abstract

The development of targeted constructs on the basis of photoluminescent nanoparticles with a high photo- and chemical stability and absorption/emission spectra in the “transparency window” of biological tissues is an important focus area of present-day medical diagnostics. In this work, a targeted two-component construct on the basis of upconversion nanophosphors (UCNPs) and anti-tumor 4D5 scFv was developed for selective labeling of tumor cells overexpressing the HER2 tumor marker characteristic of a number of human malignant tumors. A high affinity barnase : barstar (Bn : Bs) protein pair, which exhibits high stability in a wide range of pH and temperatures, was exploited as a molecular adapter providing self-assembly of the two-component construct. High selectivity for the binding of the two-component 4D5 scFv-Bn : UCNP-Bs construct to human breast adenocarcinoma SK-BR-3 cells overexpressing HER2 was demonstrated. This approach provides an opportunity to produce similar constructs for the visualization of different specific markers in pathogenic tissues, including malignant tumors.

## INTRODUCTION

The use of modular constructs based on immunoglobulin superfamily proteins for targeted drug delivery and diagnosis is the current trend in molecular medicine referred to as theranostics [1-3]. In this case, special interest is generated by the problem of developing targeted constructs based on photoluminescent nanoparticles that have targeting modules that ensure their delivery to the target cells [4]. This approach enables the development of fundamentally new, highly efficient techniques for personalized optical diagnosis. These constructs, which accumulate in target cells, can contrast these cells against a background of healthy tissues due to photoluminescent response to excitation by light of a certain wavelength. In particular, the use of photoluminescent constructs capable of targeted binding to an appropriate cell cancer marker provides the most sensitive and noninvasive early diagnosis method for cancer.

Cell cancer markers such as the protein of the family of human epidermal growth factor receptors HER2 are abundant in tumor tissues, where they serve as effective targets for the detection and therapy of cancer. HER2 is overexpressed in many tumors, including tumors of the ovarian, uterine cervix, urinary bladder, rectum, stomach, esophagus, and breast, and the level of its expression is often correlated with poor prognosis and increased resistance to chemotherapy [5]. Consequently, the development of HER2-targeted photoluminescent constructs is one of the most promising approaches in the early-molecular diagnosis of cancer. Targeted delivery is provided by the use of a targeting module of protein nature, which is part of the construct. As the HER2-targeted module, a full-length humanized monoclonal antibody, 4D5, was used. It is widely utilized in clinical practice under the trade name Herceptin® [6]. The antibody was attached to a nanoparticle using a variety of crosslinking agents or simple physical adsorption. In this study, a genetic engineering fragment of the antibody 4D5 scFv is used as a targeting module that is a single polypeptide chain in which the variable domains of light and heavy immunoglobulin chains are connected by short flexible linkers, and the constant domains are lacking [7]. The 4D5 scFv fragment, as the targeting module, attracts attention because it is also capable of effectively recognizing HER2 [8-10], but, unlike full length antibodies, it does not provide interaction with the receptors of immune system cells and complement system proteins [11].

To develop the targeted constructs, the principle of self-assembly through the system of barstar : barnase (Bs : Bn) protein adapters was suggested. It enables the combination of individual modules with different functionalities and the generation of constructs with a predetermined set of properties [12-15]. Bacterial ribonuclease Bn and its natural inhibitor Bs form a strong complex with a dissociation constant of ~ 10-^14^ M [16] and high individual stability in a wide range of pH and temperatures [17]. Furthermore, the feature of these proteins is their biotechnological producibility, and their use allows one to improve the properties of the targeted constructs. For example, Bn as a part of genetically encoded fusion proteins acts in some cases as an intramolecular chaperone providing correct folding of the composite domains comprising the targeting modules [18].

Upconversion nanophosphors (UCNPs) are inorganic photoluminescent nanoparticles whose photoluminescence is based on upconversion, which is the process of converting several photons of lower energy (longer wavelength) into a single photon with a higher energy (shorter wavelength). UCNPs are highly efficient contrasting agents with unique photoluminescence properties; they have a whole number of advantages compared to the fluorescent proteins and organic dyes traditionally used for optical diagnosis. These include their exceptional resistance to photodegradation and chemical degradation, excitation with wavelengths (typically 980 nm) falling into the “transparency window” of biological tissue, and long-term photoluminescence shifted to the shorter wavelengths, including visible and far-red light [19]. Moreover, long-term photoluminescence provides the possibility of delayed signal detection, which makes possible the elimination of tissue autofluorescence and the achievement of a significant increase in the image contrast at the individual nanoparticle level in a biological environment.

UCNP-based targeted constructs were used in a number of studies devoted to the visualization of cell and tissue structures [20], including cancer cells with HER2 overexpression. Targeting of UCNPs at HER2 by means of full-length antibodies attached to them was described in [21, 22]. In this paper, we offer a new approach to the development of targeted constructs on the basis of UCNP and 4D5 scFv specific to the HER tumor marker, by means of self-assembly through the system of Bs : Bn molecular adapters.

## EXPERIMENTAL

**Synthesis of UCNP**

Hydrophobic UCNPs in the form of NaYF_4_ crystals doped with Yb_3_^+^ and Er_3_^+^ and bearing a surface oleate anion were synthesized by the method described in [23]. Crystals of programmable size were grown from a solution of sodium salts and oleic acid in an oxygen-free atmosphere. A mixture of Y_2_O_3_ (0.78 mM), Yb_2_O_3_ (0.2 mM), and Er_2_O_3_ (0.02 mM) was refluxed in 70% trifluoroacetic acid (20 mL) for 6 h. The clear solution was then cooled to room temperature, and the solvent was evaporated. The resulting precipitate was dried under 0.1 mmHg vacuum for 3 h and triturated carefully in an agate mortar to a homogeneous state. This powder was mixed with sodium trifluoroacetate (2 mM), oleic acid (6 mL), and 1-octadecene (6 mL) at 100 °C in vacuum for 30 min.

The degased and dehydrated mixture was gradually heated to 290 °C at arate of 6 °C/min and held at this temperature for 45 min under argon. The temperature was then raised to 310 °C for 70 min. In the next step, the solution was cooled, suspended in isopropanol (130 mL), and centrifuged at 6,000 rpm for 30 min (Z206A centrifuge, Hermle, Germany). The resulting particles were washed four times in absolute ethanol and dried. The particles were then dissolved in chloroform (10 mL), precipitated with isopropanol (50 mL), and centrifuged twice at 4,000 rpm for 10 min. The desired product was dried at room temperature.

**Preparation and characterization of proteins**

The recombinant **4D5 scFv-Bn **protein consisting of Bn and 4D5 scFv connected by a flexible peptide linker was produced as previously described [7], with slight modifications.* Escherichia coli *cells of the SB536 strain [F-, WG1, Δ*fhuA *(ton Δ), Δ*hho*AB (SacII), *shh*] were transformed with the pSD4D5BnHis5 plasmid bearing the gene encoding the 4D5 scFv-Bn protein under the control of the *lac*-promoter and with the Bs gene, whose constitutive synthesis protects bacterial cells from the cytotoxic effect of Bn [24]. Transformants were grown in a nutrient YTPS broth (1% yeast extract, 1% tryptone, 150 mM NaCl, 40 mM K_2_HPO_4_, 10 mM KH_2_PO_4_, 2 mM MgCl_2_, 0.1 g/L ampicillin, pH 7.5) at 37 °C until the optical density 0.6 at 560 nm; then, β-*D*-1-thiogalactopyranoside (1 mM) was added to induce the *lac*-promoter and transformants were incubated for more 5 h. The resulting biomass was collected by centrifugation (Allegra 21R centrifuge, Beckman Coulter, USA) and sonicated on ice in a lysis buffer with 5 mM Tris-HCl, 40 mM K_2_HPO_4_, and 500 mM NaCl, pH 8.2. The resulting extract was clarified by centrifugation and filtration through a membrane filter with a pore size of 0.22 μm and loaded onto the 1 mL HiTrap column with an affinity sorbent, Ni-nitrilotriacetic acid (Ni-NTA), (GE Healthcare Worldwide, USA). To free the target 4D5 scFv-Bn protein from the Bs inhibitor, the column was washed with 8 M urea, followed by refolding of 4D5 scFv-Bn with a linear urea gradient of 8–0 M. The target protein was eluted with 225 mM imidazole, transferred into a phosphate buffer (20 mM NaCl, 6.5 mM NaH_2_PO_4_, 41 mM Na_2_HPO_4_, pH 6.5) on the desalting PD-10 column (GE Healthcare Worldwide, USA), and subjected to final purification on the 1 mL HiTrap SP-Sepharose Fast Flow cation exchange column (GE Healthcare Worldwide, USA) that was eluted with a NaCl gradient; fractions were analyzed by electrophoresis in a 12.5% PAAG. According to the electrophoretic analysis in a 12.5% PAAG, the fraction of the target 4D5 scFv-Bn protein was eluted in 275 mM NaCl.

The cysteine-free **Bs (C40/82A) **barstar mutant was isolated from *E. coli *cells of the HB101 strain [F- Δ (*gptproA) 62 leu B6 glnV44 ara-14 galK2 lacY1Δ(mcrC-mrr) rpsL20 (*Strr*) xyl-5 mtl-1 recA13*] bearing the pMT641 plasmid [7]. The bacterial culture was grown in a YTPS broth to the stationary phase, and the cells were separated by centrifugation and re-suspended in a cold lysis buffer of the following composition: 0.05 M Tris-HCl, 0.1 M NaCl, 10 mM EDTA, 10 mM dithiothreitol, pH 8.0. The cells were disrupted by sonication on ice (30% saturation with (NH_4_)_2_SO_4_), then nucleic acids were precipitated with polyethyleneimine. Proteins were precipitated from the resulting cell extract by adjusting the ammonium sulfate concentration to 70% saturation. The protein precipitate was dissolved in a buffer (0.1 M Tris-HCl, 10 mM EDTA, 10 mM dithiothreitol, pH 8.0) and size-fractionated on the Sephadex G-100 Super- Fine (C16/100) column equilibrated with a buffer: 0.02 M Tris-HCl, 0.02 M NaCl, 2 mM EDTA, 2 mM dithiothreitol, 0.05% Tween-20, pH 8.0. Final purification of Bs was performed on the 1mL HiTrap Q-Sepharose Fast Flow anion exchange column (GE Healthcare Worldwide, USA) equilibrated with a buffer: 0.2 M Tris-HCl, 2 mM dithiothreitol, 10% glycerol, pH 8.0. The target protein was eluted with a NaCl gradient; the fractions were analyzed by electrophoresis in 17% PAAG.

**Ribonuclease activity of the recombinant 4D5 scFv-Bn protein**

The ribonuclease activity of the recombinant 4D5 scFv- Bn protein was determined by the acid-insoluble RNA precipitate method [25]. 40 μL of the analyzed protein solution with a concentration of 30 to 0.015 nM in a 0.125 M Tris-HCl buffer, p H 8.5, was mixed with 160 μL of a yeast RNA solution (2 mg/mL) and incubated at 37 °C for 15 min. The RN Ase reaction was quenched with 6% HClO3 (200 μL), and the mixture was kept at 2 °C for 15 min. Unreacted RNA was separated by centrifugation. The concentration of the released nucleotides, which was directly proportional to the RNAse activity of the studied protein, was determined by optical absorption (OD_260_).

To assess the binding of the Bs : Bn pair, different Bs dilutions were added to the Bn solution at a known concentration and RNAse activity was measured as described above. In the latter case, the Bs concentration was inversely proportional to OD_260_.

**Affinity of the 4D5 scFv-HER2/neu protein to the HER2 receptor**

Affinity of the 4D5 scFv-HER2/neu protein to the HER2 receptor was assessed using polyclonal rabbit anti-human IgG antibodies. The p185^HER2-ESD^ antigen (a recombinant protein that is an extracellular domain of the HER2 receptor) in a buffer (0.1 M Na_2_CO_3_, 0.1 M NaHCO_3_, pH 9.2) was added into polystyrene flat bottom 96- well plates in the amount of 8 and 16 ng/well. After adsorption of the antigen for 1 h, the plates were washed with PBS and the unsaturated surface binding sites were blocked with a 5% solution of milk powder (Tesco, UK) in PBS, pH 7.4. The 4D5 scFv-Bn protein solution in PBS with 0.1% Tween-20 at different concentrations, beginning with 5 nM, was added to the wells and incubated for 1 h on a shaker, then washed. To detect the immobilized 4D5 scFv-Bn protein, the plates were treated with polyclonal rabbit anti-human IgG antibodies, followed by goat anti-rabbit IgG antibodies conjugated to horseradish peroxidase with washes between stages. For colorimetric measurement, the wells were added with 0.04% 1,2-diaminobenzene (Sigma-Aldrich, Germany) with 0.06% H_2_O_2_ in a citrate buffer (7.3 g/L citric acid, 11.86 g/L Na_2_HPO_4_∙2H_2_O, pH 5). The reaction was stopped by adding 50 μL of 2 M H_2_SO_4_, and OD_450_ was measured, and on a tablet spectrophotometer (Stat- Fax-2100, Awareness Technology, USA). The affinity constant, *K_a_*, was calculated as described in [26], taking into account the monovalency of the studied mini-antibody according to the following equation:

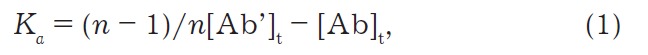



where [Ab’]_t_ and [Ab]_t_ are total concentrations of the mini-antibody in wells with the values OD_450_’ and OD_450_ treated with the antigen at concentrations of [Ag’] (8 ng) and [Ag] (16 ng), respectively,

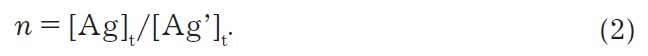



**Preparation of UCNP bioconjugates**

UCNPs synthesized as described above were coated with an amphiphilic alternating copolymer of maleic anhydride and 1-octadecene (PMAO, Sigma-Aldrich, Germany) as described in [27] with minor modifications. To create a PMAO shell around the UCNP particles and to form cross links, 1,6-diaminohexane (Serva, Germany) was added. To attach the biomolecules to UCNP, the surface carboxyl groups of the resulting PMAO shell were activated with an excess of cross-linkers, 1-ethyl-3-(3-dimethylaminopropyl)carbodiimide hydrochloride (EDC) and N-hydroxysulfosuccinimide (sulfo-NHS) (Sigma-Aldrich, Germany), in a cold buffer with additional sonication. The resulting nanoparticles were then washed from the unreacted cross-linkers by centrifugation at 4 °C, re-suspended in a cold Bs protein solution, and incubated overnight for the attachment of Bs. Unbound Bs molecules were washed out in three cycles of centrifugation/re-suspension. The resulting nanoparticles were stored in PBS.

**Transmission Electron Microscopy (TEM)**

UCNP and UCNP-PMAO were dissolved in *n*-hexane and water, respectively, sonicated, and laid on copper TEM grids (300 mesh) coated with a 0.3% Pioloform® solution (Wacker Polymer Systems, Burghausen, Germany). Then the grids were dried at room temperature in a desiccator overnight and microscoped on a Philips CM10 TEM device (Philips, The Netherlands). The ImageJ software was used to analyze the UCNP fractional composition.

**Infrared Spectroscopy**

Free PMAO was carefully triturated with KBr in a mortar and compressed to form tablets. PMAO-modified UCNPs were dried using a Savant SpeedVac concentrator (France), then they were triturated with KBr and compressed to form tablets. IR spectra were recorded on a Varian 3100 spectrophotometer (USA).

**Detection of the UCNP-PMAO emission spectra**

The UCNP-PMAO powder was placed in a sample holder and irradiated by a laser with a wavelength of 978 nm through a multimodal optical fiber. An emission signal, passed through an emission filter with a bandwidth to 842 nm (Semrock, USA), was recorded in the transmitted light on a calibrated spectrophotometer (Ocean Optics, USA).

**Cell labeling**

Human breast adenocarcinoma SK-BR-3 cells and Chinese hamster ovary CHO-K1 cells (American Type Culture Collection, USA) were cultured in a RPMI- 1640 culture medium (HyClone, USA) supplemented with *L*-glutamine and 10% fetal bovine serum (Hy- Clone, USA). The cells were seeded onto 8-well glass slides at a concentration of 3 × 104 cells/mL and cultured at 37 °C in a CO_2_ incubator (5% CO_2_) for 24 h. The cells were inactivated by addition of 1% formaldehyde to prevent nonspecific internalization. To prevent nonspecific binding of the particles, the cover glasses were treated with 1% bovine serum albumin (BSA) (Bio-Rad, USA) in PBS for 1 h. Then, to create cell-surface sites for specific binding of an imaging agent, Bs-UCNP, the recombinant 4D5 scFv-Bn protein solution in PBS containing 0.1% BSA and 0.1% Tween-20 was laid on the glasses and incubated for 1 h. The cells were then washed with PBS and treated with a colloidal Bs-UCNP solution (100 μg/mL) for 20 min. This time was enough to complete the formation of 4D5 scFv-Bn : Bs-UCNP complexes due to the exceptionally high affinity constant of the Bs : Bn pair ~ *K_d_*10^-14^ M.

The cells were then washed several times from unbound Bs-UCNP, fixed in a 4% formaldehyde solution in PBS, and covered with a cover glass. In order to demonstrate that binding of UCNP is not a result of the nonspecific 4D5 scFv-Bn protein adsorption on the glasses, CHO cells were used as a negative control.

**Photoluminescence cell microscopy**

Photoluminescence cell microscopy was performed on an Olympus IX70 epi-luminescence inverted microscope (Japan) with excitation at 978 nm by a diode laser (LD980-01CW, CXCH-Photonics, China). To visualize the cells in visible light, a dry lens × 50, NA 0.45 (Olympus, Japan) was used.

## RESULTS AND DISCUSSION

**Fig. 1:**
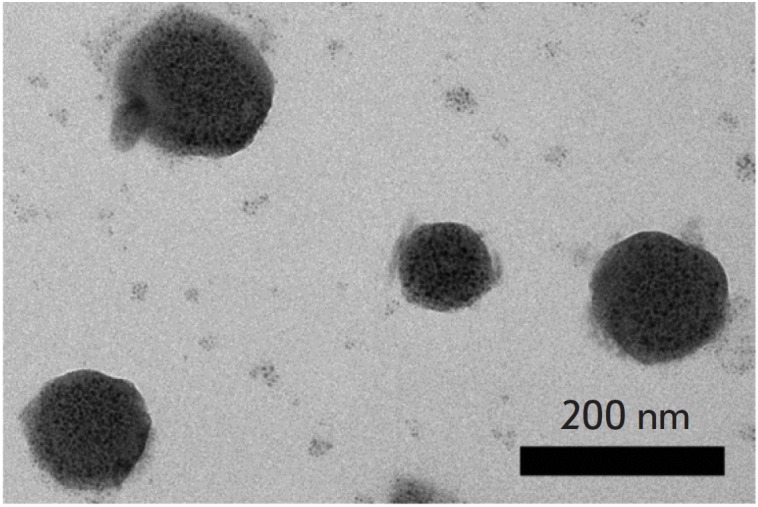
Transmission electron microscopy imaging of UCNP- PMAO

Hydrophobic UCNPs in the form of NaYF_4_ crystals doped with Yb_3_^+^ and Er_3_^+^ and bearing oleate ani ons on their surface were synthesized by the method described previously [23]. In order to impart hydrophilicity, the particles were further coated with the molecules of an alternating copolymer, poly(maleic anhydride-1-octadecene) (PMAO), interconnected by means of 1,6-diaminohexane (Serva, Germany). When the UCNP-PMAO particles are transferred from an organic solvent into water, the anhydride ring opens to form a carboxyl group exposed to the solution, which ensures the solubility of UCNP in water [27]. The UCNP-PMAO hydrodynamic diameter measured by dynamic light scattering was 130 ± 20 nm. *Figure 1 *presents an image obtained by a transmission electron microscope of nanoparticles with the polymicellar PMAO structure on the UCNP surface. According to measurements using the calibrated integrating sphere, maximum efficiency of nanoparticle upconversion was achieved with a laser excitation power density of ~ 60 W/cm^2^ and amounted to 1.2%.

**Fig. 2:**
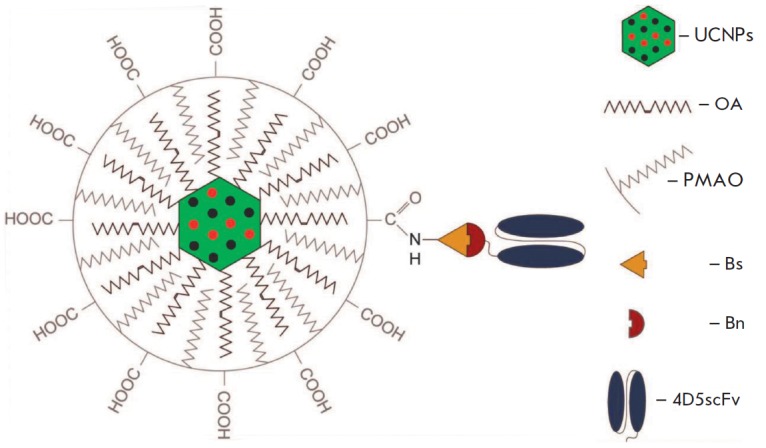
Structural diagram of the targeted UCNP-PMAOBs : 4D5 scFv-Bn construct. UCNPs – upconversion nanophosphors, OA – oleate anion, PMAO – poly(maleic anhydride-*alt*-1-octadecene) amphiphilic polymer, Bs – barstar, Bn – barnase, 4D5 scFv – variable fragment of anti-HER2-antibody 4D5

Evaluation of the potential of UCNPs as agents for the optical imaging of target cells was carried out *in vitro *using human breast adenocarcinoma SK-BR-3 cells overexpressing the HER2 surface tumor marker. For this purpose, a targeted, two-component construct was developed that comprised contrasting and targeting modules capable of assembling by means of a system of molecular Bs : Bn adapters, as shown in *Figure 2*. The contrasting module was produced by conjugation of mutant Bs C40/82A with carboxyl groups of UCNPPMAO using the crosslinking reagents 1-ethyl-3-(3- dimethylaminopropyl) carbodiimide hydrochloride and N-hydroxysuccinimide. The resulting conjugates retained the non-aggregated state and photoluminescence parameters. The targeting module capable of highly efficient binding to the external domain of the HER2 receptor on the tumor cell surface was represented by the recombinant fusion 4D5 scFv-Bn protein described in [7]. It was produced by attachment of Bn to the C-terminal segment of the 4D5 scFv via a flexible peptide linker. Both polypeptides were proven [7] to preserve their functional properties – the ability to specifically recognize the HER 2 receptor (4D5 scFv) and the ability to bind Bs with high affinity (Bn).

**Fig. 3:**
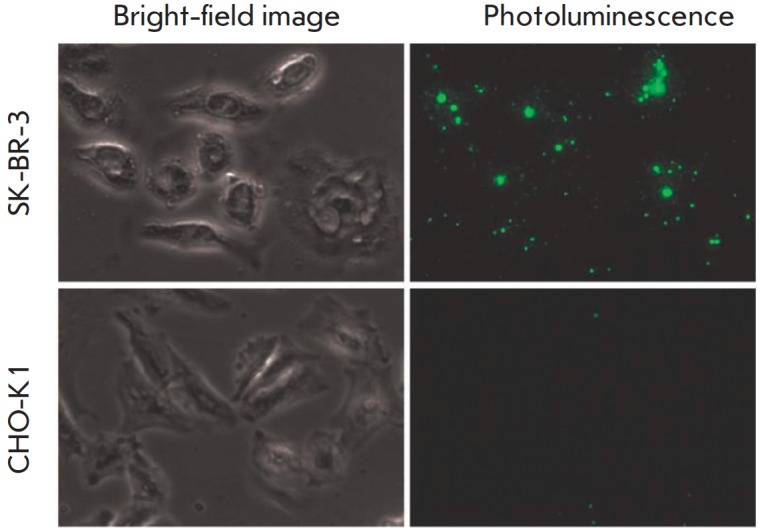
Photoluminescence microscopy imaging of SK-BR-3 cells overexpressing the HER2 tumor marker, and CHO cells (negative control) after treatment with UCNP-PMAO-Bs and 4D5 scFv-Bn

Highly sensitive visualization of SK-BR-3 cells using the described two-component construct was realized via two-stage delivery. To recognize the HER2 receptor, cells, grown on a scaffold and fixed with formaldehyde, were treated with the targeting 4D5 scFv-Bn module. Then, for visualization purpose, the cells were added with a contrasting Bs-UCNP module that provided optical detection through binding to Bn, which was part of the 4D5 scFv-Bn module immobilized on HER2. After incubation, excess Bs-UCNP was removed by thorough washing of cells with a phosphate buffer. Chinese hamster ovary CHO cells lacking HER 2 were used as a negative control. Based on photoluminescence microscopy of SK-BR-3 and CHO cells treated sequentially with the 4D5 scFv-Bn and Bs-UCNP modules, with the excitation of luminescence at 978 nm (*Figure 3*), it was demonstrated that the produced two-component 4D5 scFv-Bn : Bs-UCNP construct selectively binds to SK-BR-3 cells overexpressing the HER2 receptor and does not bind to control CHO cells lacking HER2. The total luminescence signal from the surface of tumor SK-BR-3 cells was 10 times higher than the signal from the surface of the control CHO cells.

## CONCLUSIONS

The produced hybrid constructs consisting of targeting biopolymer molecules and inorganic photoluminescent nanocrystals are capable of highly specific visualization of the cancer marker on tumor cells. These nanoconstructs can serve as promising carriers for the targeted delivery of a wide variety of cytotoxic and imaging agents, which creates new opportunities for a highly accurate molecular diagnosis and effective therapy of tumor diseases. An important advantage of the UCNP-based constructs [28] is the possibility of their detection deep in the living tissue, which determines their particular potential for personalized optical diagnosis of malignant tumors.

## Abbreviations

EDC: 1-ethyl-3-(3-dimethylaminopropyl)carbodiimide
HER2: human epidermal growth factor receptor 2
PBS: phosphate buffered saline
scFv: single chain variable antibody fragment
sulfo-NHS: N-hydroxysulfosuccinimide
Bn: barnase
Bs: barstar
BSA: bovine serum albumin
UCNPs: upconversion nanophosphors
PAAG: polyacrylamide gel
PMAO: poly(maleic anhydride-alt-1-octadecene) amphiphilic polymer
TEM: transmission electron microscopy
